# Lived Experiences of Coping With Type 1 Diabetes Among Palestinian Youth: A Qualitative Study

**DOI:** 10.1155/pedi/9014326

**Published:** 2025-11-02

**Authors:** Buthaina Nazzal, Lobna Harazni, Ibrahim Aqtam, Rasmieh Anabtawi, Ahmad Ayed

**Affiliations:** ^1^Department of Nursing, Faculty of Nursing, Arab American University, Jenin, State of Palestine; ^2^Department of Nursing, Ibn Sina College for Health Professions, Nablus University for Vocational and Technical Education, Nablus, State of Palestine

**Keywords:** adolescence, content analysis, coping, Palestine, self-care, social support, stigma, type 1 diabetes, West Bank

## Abstract

**Background:**

Type 1 diabetes mellitus (T1DM) in adolescents requires continuous self-care and emotional adjustment. Palestinian youth with T1DM face unique challenges within the context of the West Bank healthcare system, where political instability, resource limitations, and cultural factors create additional barriers to optimal diabetes management. These youth experience heightened social stigma, cultural dietary pressures, and restricted access to specialized healthcare services.

**Aim:**

To explore the lived experiences, challenges, and coping strategies of Palestinian youth with T1DM in the West Bank.

**Methods:**

This qualitative content analysis study was conducted from May–June 2025. Eighteen Palestinian adolescents (12–18 years) with T1DM were recruited through purposive sampling from West Bank diabetes clinics. Participants were approached directly at clinics by trained researchers who explained the study's voluntary nature and ensured understanding that participation would not affect clinical care. For minors under 16, guardians were present during consent, and all participants provided appropriate consent/assent. Data were collected via semi-structured interviews (45–90 min) and analyzed using conventional content analysis methods. Credibility was established through prolonged engagement, peer debriefing, and member checking with five participants.

**Results:**

Four main themes emerged from the analysis: challenges from social and cultural pressures, including stigma and dietary expectations; reliance on support systems involving family, peers, and healthcare access; emotional and spiritual coping through resilience, anxiety management, and faith-based strategies; daily self-management focused on insulin routines and food planning. Participants described significant barriers, including limited healthcare resources, medication shortages, cultural food pressures during religious fasting periods, and social misunderstandings about diabetes. Despite these challenges, many demonstrated remarkable resilience through family support, religious coping, and adaptive self-care routines.

**Conclusion:**

Palestinian adolescents with T1DM navigate complex challenges that extend beyond medical management to include cultural, social, and political barriers. Their coping strategies are deeply embedded in family support systems and religious faith. The findings highlight the critical need for culturally responsive, family-centered diabetes care that addresses both medical and psychosocial needs while considering the unique context of life in the West Bank.

## 1. Introduction

Type 1 diabetes mellitus (T1DM) is a chronic disease that destroys insulin-producing β-cells in the pancreas. The current paradigm in this disease's etiopathogenesis points toward the interplay of genetic and environmental factors [1]. Palestinian adolescents with T1DM face distinct challenges within the context of the West Bank healthcare system, where ongoing sociopolitical instability creates unique barriers to diabetes management. These youth must navigate complex cultural expectations while managing a chronic condition that requires constant vigilance and medical support.

### 1.1. Context of Diabetes Care in the West Bank

The healthcare system in the West Bank operates under challenging conditions characterized by resource limitations, movement restrictions, and fragmented service delivery. Diabetes care is primarily provided through Ministry of Health clinics, private facilities, and NGO-supported centers, but access varies significantly by geographic location and economic status. The Palestinian National Diabetes Program (PNDP) has worked for over two decades to improve diabetes care, yet systemic challenges persist, including inconsistent medication supplies, limited specialist availability, and barriers to continuous care due to political restrictions [2, 3].

Despite advances in treatments and high-quality care, patients still require lifelong commitment and personal responsibility for their health and behaviors, despite the complexity of the condition [4]. Type 1 diabetes-related distress develops over time, especially in adults diagnosed since childhood [5]. For Palestinian youth, this distress is compounded by environmental stressors, including political tension, economic hardship, and social stigma that may not be present in other contexts.

Coping with T1DM among Palestinian adolescents is influenced by multiple intersecting factors, including resource availability, family and community support, cultural awareness about diabetes, socioeconomic constraints, and the broader political environment [6]. These factors create a unique constellation of challenges that require culturally informed understanding and intervention approaches.

The prevalence of T1DM among the Palestinian population was 3.4% [7]. Modern diets, high carbohydrates, and sedentary lifestyles contribute to the problem [8]. Political obstacles and insufficient resources further exacerbate psychological distress among Palestinian children [8].

A critical issue is underdiagnosis, particularly among children. An estimated 10.4% of children who develop T1D in Palestine remain undiagnosed, highlighting gaps in screening and healthcare access. This aligns with broader challenges in resource-limited settings, where delayed diagnoses exacerbate complications, like hypoglycemia. Microvascular complications affect 34.4% of Palestinian individuals with diabetes, with renal disease, retinopathy, and neuropathy linked to suboptimal glycemic management and limited preventive care [9].

In Gaza, conflict exacerbates these challenges. 79.3% of children with T1D cannot access adequate care during periods of violence, leading to medication shortages, food insecurity, and frequent hospitalizations. Inadequate glycemic management in these conditions increases the risks of acute complications and long-term morbidity [8]. Nationally, diabetes prevalence among adults aged 20–79 is reported at 9.2% (2021), though anecdotal estimates suggest rates as high as 18%–21% due to undiagnosed cases and rising obesity.

Efforts to address these issues include the PNDP, which aims to upgrade clinics, implement gestational diabetes protocols, and train healthcare providers [2]. However, systemic barriers, such as fragmented medical records and reliance on clinic-based data, complicate accurate surveillance and intervention [10, 11].

Recent studies have shown that adherence to diabetes management is crucial among Palestinian patients. Additionally, children living with type 1 diabetes face unique challenges in the West Bank. Societal norms significantly influence the daily lives of children with diabetes and their families. The impact of war on diabetes management in Gaza has also been explored. Furthermore, the PNDP has worked to combat diabetes for over two decades [2, 3].

The war, restrictions on movement, and economic hardship in Palestine limit access to critical diabetes care services, like insulin, glucose monitoring devices, and specialized consultations that are essential for good health [3]. Moreover, in Palestine, adolescents with T1DM may face added psychological stressors, including anxiety, depression, and stigma, which can complicate their disease management and emotional health.

Young people from Palestine are affected by social and familial support regarding diabetes self-management and more. A strong family involvement and peer support could improve treatment adherence and strengthen resilience to illness [12]. In a low-resource setting, families face financial burdens as well as limited educational resources. It makes it difficult for young patients to develop effective coping skills. Given these multifaceted challenges, understanding how Palestinian youth cope with T1DM is essential for developing culturally appropriate and contextually relevant interventions that can improve both health outcomes and quality of life in this vulnerable population.

Due to those challenges, it is necessary to investigate the coping strategies for Palestinian youth with T1DM to assist in developing appropriate interventions in the Palestinian context. It can be helpful to highlight the essential factors of these coping behaviors to the healthcare providers, policymakers, or community members, for example, by ensuring support, access to education, and holistic diabetes care in Palestine. This research examined the coping strategies of Palestinian children and youth with T1DM and looked at their psychosocial adjustment, social support, and access to healthcare.

### 1.2. Research Aim and Questions

This study aimed to explore the lived experiences, challenges, and coping strategies of Palestinian youth with T1DM in the West Bank. The research was guided by the following questions:

1. What are the primary challenges affecting diabetes management among Palestinian youth with T1DM?
2. What coping strategies do these young people employ?
3. What barriers affect their ability to cope effectively with their condition?
4. How do cultural and social factors influence their diabetes management experiences?

## 2. Methodology

### 2.1. Study Design

This study employed a qualitative content analysis approach using conventional content analysis methods to explore the experiences of Palestinian youth with T1DM. Content analysis was chosen as the most appropriate method for this research as it allows for systematic examination of communication content to identify patterns, themes, and meanings within participants' experiences [13]. The reporting of this qualitative study adheres to the Consolidated Criteria for Reporting Qualitative Research (COREQ) checklist (Supporting Information 1: S1). Unlike phenomenological approaches that focus on the essence of lived experience, content analysis enables researchers to describe and quantify specific phenomena within the data while maintaining the richness of participants' voices.

The choice of content analysis was particularly suitable for this study because it allows for both inductive theme development from the data and practical application of findings to healthcare practice and policy development.

### 2.2. Research Questions

The study was guided by the following specific research questions:

1. What are the primary challenges affecting diabetes management among Palestinian youth with T1DM?
2. What are the coping strategies employed by those young people?
3. What are the barriers affecting their ability to cope effectively?
4. How do cultural and social factors influence their diabetes management and healthcare access experiences?

### 2.3. Sample and Sampling

Purposive sampling with maximum variation was employed to recruit participants who could provide rich and diverse information about diabetes management experiences among Palestinian youth. Inclusion criteria required participants to be Palestinian youth aged 12–18 years, diagnosed with T1DM for at least 6 months, able to communicate in Arabic, and willing to participate with appropriate consent or assent. Exclusion criteria included cognitive impairments that could affect the ability to participate, acute medical complications requiring hospitalization, and non-Palestinian nationality.

Participants were recruited directly from diabetes clinics in Nablus, Jenin, Ramallah, and Hebron. Trained researchers approached eligible participants during routine clinic visits, explained the study's voluntary nature, and ensured understanding that participation would not affect their clinical care. No incentives were provided for participation. For participants under 16 years of age, guardians were present during the consent process, and both parental consent and participant assent were obtained.

Sample size was determined based on information power principles [14], considering the study's specific aim, sample specificity, theoretical foundation, quality of dialog, and analysis strategy. Sample size determination was guided by the principle of data saturation, which was reached by the fifteenth interview. To confirm saturation, three additional interviews were conducted, resulting in a total of 18 participants.

### 2.4. Data Collection

A semi-structured interview guide was developed based on comprehensive literature review and consultation with experts in pediatric diabetes care and qualitative research methodology. The guide was pilot tested with two participants, whose data were not included in the final sample, to assess clarity and relevance. The questions were open-ended to encourage narrative, detailed responses, and probing questions were used to explore participants' experiences and perspectives in greater depth. For expanded details on the participant demographics and the thematic overview, see Supporting Information 2: S2.

### 2.5. Interview Conduct

Interviews lasted between 45 and 90 min, with an average duration of 62 min. They were conducted in Arabic by trained researchers to ensure cultural and linguistic appropriateness. All interviews were audio-recorded using digital recorders with participants' informed consent. Field notes were systematically recorded during and immediately after each interview to capture non-verbal cues, contextual information, and interviewer observations that could enhance data interpretation.

Member checking was conducted with five participants through follow-up phone interviews 2–4 weeks after the initial interview, during which participants were presented with preliminary interpretations of their individual interviews and asked to verify accuracy and provide additional clarifications.

### 2.6. Data Analysis Framework

Data analysis followed conventional content analysis procedures [13]:

Step 1: Immersion: all transcripts were read multiple times by the research team to achieve overall familiarity with the data. Step 2: Initial coding: open coding was conducted to identify initial concepts and categories. Step 3: Categorization: related codes were grouped into broader categories. Step 4: Abstraction: categories were further abstracted into main themes. Step 5: Validation: themes were validated through team discussion and member checking

### 2.7. Coding Process

Data analysis followed established content analysis procedures. Initial coding was conducted independently by two researchers (Buthaina Nazzal and Lobna Harazni) to enhance analytical reliability and reduce potential bias. Codes and categories were developed inductively from the data without predetermined theoretical frameworks. Intercoder agreement was assessed through discussion and consensus-building rather than statistical measures, as appropriate for qualitative content analysis. Any discrepancies in interpretation were resolved through discussion and consultation with the broader research team. ATLAS.ti software was used to facilitate data management and support the coding process.

### 2.8. Researcher Reflexivity and Positioning

The research team maintained reflexivity throughout the study to acknowledge and address potential influences on data collection and analysis. The primary researcher (Buthaina Nazzal), a registered nurse with pediatric diabetes care experience, engaged in reflective journaling before, during, and after each interview. The team held regular debriefing sessions to discuss emerging findings, challenge interpretations, and ensure diverse perspectives were considered. Researcher assumptions and prior experiences were explicitly documented and discussed within the team to enhance transparency and minimize bias.

### 2.9. Credibility and Trustworthiness

Credibility was established through multiple strategies:

  Prolonged engagement: 6-month fieldwork period allowing development of rapport and deep understanding of context.
  Member checking: follow-up interviews with five participants to verify interpretations.
  Peer debriefing: regular team meetings to review data and challenge interpretations.
  Triangulation: multiple data sources, including interviews and field notes.
  Thick description: Detailed contextual information provided to support transferability.

Dependability was ensured through detailed documentation of all methodological decisions and consistent application of analysis procedures across all data. An audit trail was maintained documenting the research process from conception through final reporting.

### 2.10. Ethical Considerations

Ethical considerations were carefully addressed through enhanced informed consent procedures. Written information sheets were provided to participants at least 48 h before the interviews to allow sufficient time for review. Study procedures and potential risks were explained verbally to ensure clear understanding. Participants were given ample opportunity to ask questions before consenting. The voluntary nature of participation and the right to withdraw at any time were clearly communicated. For minors, separate assent forms were used, and for participants under 16 years of age, a guardian was present during the consent process to support informed decision-making.

Data protection measures were rigorously implemented to ensure participant confidentiality and data security. All data were de-identified immediately after collection to remove any personal identifiers. Data were stored securely in password-protected systems with access restricted solely to authorized research team members.

### 2.11. Findings

#### 2.11.1. Participant Demographics

Table 1 presents the demographic characteristics of the 18 study participants.

#### 2.11.2. Thematic Analysis Results

The analysis revealed four main themes that encompass both the challenges faced and coping strategies employed by Palestinian youth with T1DM. These themes represent the complex interplay between individual adaptation and broader contextual factors that shape their diabetes management experiences.

#### 2.11.3. Theme 1: Navigating Social and Cultural Pressures (All Participants, *n* = 18)

This theme encompasses the various social and cultural challenges participants face in managing their diabetes within Palestinian society. All participants described experiencing significant social and cultural pressures related to their T1DM diagnosis.

#### 2.11.4. Subtheme 1.1: Confronting Stigma and Misconceptions

Many participants reported experiencing social stigma rooted in widespread misconceptions about diabetes within their communities. This stigma manifested in various forms, from social avoidance to discriminatory attitudes that significantly impacted participants' psychological well-being and social interactions.

*“They think diabetes is contagious, and they don't come near me. It makes me feel like I am different and sometimes I just want to hide my condition.”* (Participant 3, female, 14 years)

*“Some people believe it's a punishment or that I did something wrong to get it. It's hard to deal with their judgment when you know they don't understand.”* (Participant 7, male, 16 years)

*“At school*, *some teachers treat me like I'm fragile or broken. They don't let me participate in sports or activities*, *even when I feel fine.”* (Participant 11, female, 15 years)

#### 2.11.5. Subtheme 1.2: Negotiating Cultural Food Practices and Dietary Requirements

Cultural practices surrounding food and hospitality presented ongoing challenges for diabetes management. Participants frequently described difficult situations during family gatherings, religious celebrations, and social events where refusing traditional foods was seen as culturally inappropriate.

*“At family gatherings, everyone insists you eat. It's rude to refuse, but I know I shouldn't eat all that sugar. It's a constant battle between being respectful and taking care of my health.”* (Participant 12, male, 17 years)

*“During Ramadan, managing my insulin while fasting is so complicated. My family worries, but I want to participate like everyone else. The doctor says it's possible, but it requires very careful planning.”* (Participant 8, female, 16 years)

*“My grandmother always tries to give me traditional sweets during Eid*, *saying 'just a little won't hurt.' She means well*, *but she doesn't understand how it affects my blood sugar.”* (Participant 5, male, 13 years)

#### 2.11.6. Subtheme 1.3: Experiencing Social Isolation and Exclusion

Many participants described feeling excluded from social activities and peer relationships due to their diabetes management needs. This isolation was both externally imposed and self-imposed as participants sometimes withdrew to avoid difficult situations.

*“I can't stay late with my friends because I need to take my insulin on schedule. Sometimes I just don't go out anymore because it's easier than explaining everything.”* (Participant 14, female, 17 years)

*“Friends don't understand why I need to check my blood sugar or why I can't eat certain foods. They think I'm being difficult or trying to get attention.”* (Participant 9, male, 15 years)

#### 2.11.7. Theme 2: Building and Utilizing Support Systems (All Participants, *n* = 18)

This theme highlights the critical role of various support systems in helping participants manage their diabetes and cope with associated challenges. All participants emphasized the importance of family, healthcare providers, and peers in their diabetes journey.

#### 2.11.8. Subtheme 2.1: Relying on Family Support and Involvement

Family members emerged as the primary source of both practical and emotional support for all participants. Parents and siblings played crucial roles in daily diabetes management tasks and provided essential emotional encouragement.

*“My mom is my biggest supporter. She reminds me to take my insulin and helps me choose healthy foods. I couldn't do it without her constant support and understanding.”* (Participant 1, female, 16 years)

*“My whole family learned about diabetes when I was diagnosed. My dad comes with me to doctor appointments*, *and my sisters help me remember my medication times. We're all in this together.”* (Participant 4, male, 14 years)

*“When I feel discouraged or frustrated with my diabetes, my parents always remind me that I'm strong and capable. Their belief in me helps me keep going.”* (Participant 6, female, 15 years)

#### 2.11.9. Subtheme 2.2: Accessing Healthcare and Professional Guidance

Participants described varied experiences with healthcare access, highlighting both positive relationships with healthcare providers and significant systemic barriers to consistent care.

*“My doctor at the clinic is very knowledgeable and patient. She explains everything clearly*, *and I trust her advice. But sometimes it's hard to get appointments*, *especially when there are closures or checkpoints.”* (Participant 10, male, 16 years)

*“The nurses at the diabetes center taught me how to count carbohydrates and adjust my insulin. This education changed everything for me - I finally felt like I could manage my diabetes independently.”* (Participant 15, female, 17 years)

*“Sometimes we run out of insulin at the clinic*, *and my family has to buy it from private pharmacies. It's very expensive*, *and we worry about not having enough.”* (Participant 17, male, 15 years)

#### 2.11.10. Subtheme 2.3: Finding Peer Support and Shared Understanding

Connections with other youth living with diabetes provided unique validation and practical support. However, many participants expressed desire for more opportunities to connect with peers facing similar challenges.

*“I met another teenager with diabetes at the clinic*, *and we became friends. Talking to someone who really understands what I go through makes me feel less alone.”* (Participant 13, female, 16 years)

*“I wish there were support groups for young people with diabetes. Sharing experiences and tips with others would be so helpful.”* (Participant 18, male, 17 years)

#### 2.11.11. Theme 3: Developing Emotional and Spiritual Coping Strategies (17 Participants, 94.4%)

This theme encompasses the various psychological and spiritual approaches participants used to cope with the emotional challenges of living with diabetes.

#### 2.11.12. Subtheme 3.1: Building Resilience and Acceptance

Many participants demonstrated remarkable resilience in adapting to their condition and developing a sense of acceptance that allowed them to move forward with their lives.

*“At first*, *I was very sad and angry about having diabetes. But then I realized that I have to live with it*, *and feeling sorry for myself won't help. I learned to be strong and take responsibility for my health.”* (Participant 4, male, 14 years)

*“Diabetes is part of who I am now*, *but it doesn't define me. I don't let it stop me from pursuing my dreams - I just plan more carefully and take better care of myself.”* (Participant 11, female, 15 years)

#### 2.11.13. Subtheme 3.2: Managing Anxiety and Fear through Practical Strategies

Participants described various approaches to managing diabetes-related anxiety, particularly fears about hypoglycemia and long-term complications.

*“I always worry about my blood sugar dropping when I'm alone or at school. I check it frequently and always carry glucose tablets. Being prepared helps me feel more secure.”* (Participant 14, female, 17 years)

*“Sometimes I get scared thinking about complications in the future*, *but then I focus on taking good care of myself today. My doctor says that good control now prevents problems later.”* (Participant 8, female, 16 years)

#### 2.11.14. Subtheme 3.3: Drawing Strength From Faith and Spiritual Practices

For many participants, religious faith and spiritual practices provided significant comfort, strength, and a framework for understanding and accepting their condition.

*“My faith helps me a lot. I believe Allah will give me strength to deal with this challenge*, *and this belief brings me peace and comfort.”* (Participant 2, male, 13 years)

*“When things are difficult with my diabetes*, *I turn to prayer. It helps me feel less alone and gives me hope that everything will be okay.”* (Participant 16, female, 18 years)

*“I see my diabetes as a test from God*, *and I try to be patient and grateful for all the good things in my life. This perspective helps me cope with the daily challenges.”* (Participant 7, male, 16 years)

#### 2.11.15. Theme 4: Mastering Daily Self-Care and Management Routines (All Participants, *n* = 18)

This theme describes how participants adapted to and managed the complex daily requirements of diabetes care, including medication adherence, dietary management, and blood glucose monitoring.

#### 2.11.16. Subtheme 4.1: Establishing Insulin Routines and Medication Adherence

All participants described the central role of insulin management in their daily lives and the strategies they developed to maintain adherence despite various challenges.

*“Taking multiple injections every day was difficult at first*, *but now it's become a habit. I set phone alarms to remind me*, *and I always carry my insulin pen with me.”* (Participant 13, female, 16 years)

*“During school hours*, *I have to go to the nurse's office for my insulin. Sometimes I feel embarrassed*, *but I know it's necessary for my health.”* (Participant 9, male, 15 years)

*“Managing insulin during Ramadan requires very careful timing and planning. I work with my doctor to adjust my doses so I can fast safely like other Muslims.”* (Participant 8, female, 16 years)

#### 2.11.17. Subtheme 4.2: Adapting Dietary Habits and Meal Planning

Participants described significant changes to their eating habits and the development of careful meal planning strategies to maintain optimal blood glucose levels.

*“I had to completely change how I think about food. Now I read every label*, *count carbohydrates*, *and plan my meals ahead of time. It was hard at first*, *but now it's second nature.”* (Participant 18, male, 17 years)

*“My mother learned to cook differently for the whole family. We eat more vegetables and less rice and bread. It's healthier for everyone*, *not just me.”* (Participant 3, female, 14 years)

*“Eating out with friends is challenging because I need to know what's in the food. I often eat before going out so I'm not tempted by foods that will spike my blood sugar.”* (Participant 12, male, 17 years)

## 3. Discussion

This study provides important insights into the complex experiences of Palestinian youth living with T1DM in the West Bank. The findings reveal that diabetes management for these young people extends far beyond medical care to encompass navigation of cultural expectations, social barriers, and systemic healthcare challenges unique to the Palestinian context.

### 3.1. Key Findings in Context

The identification of four main themes—navigating social and cultural pressures, building support systems, developing coping strategies, and mastering self-care routines—demonstrates the multifaceted nature of diabetes management among Palestinian youth. These findings align with international literature on adolescent diabetes management while highlighting unique contextual factors specific to the Palestinian experience.

The theme of social and cultural pressures extends existing research by providing specific examples of how Palestinian cultural norms around food, hospitality, and religious practices create particular challenges for diabetes management. The pressure to participate in communal eating during religious celebrations and family gatherings, combined with widespread misconceptions about diabetes, creates a complex social environment that these youth must navigate daily.

Family support emerged as a critical factor in successful diabetes management, consistent with research emphasizing the importance of family involvement in adolescent chronic illness management [12]. However, our findings highlight how family support in the Palestinian context is intensified by external pressures and resource limitations, making family systems even more crucial for successful outcomes.

The integration of religious and spiritual coping strategies represents a significant finding that reflects the cultural values of Palestinian society. Faith-based coping appeared to provide not only emotional comfort but also a framework for understanding and accepting diabetes as part of life's challenges, consistent with research on religious coping in chronic illness [15, 16].

### 3.2. Cultural and Contextual Considerations

The challenges described by participants reflect broader issues within the West Bank healthcare system, including medication shortages, limited specialist availability, and access barriers due to political restrictions. These systemic issues compound the typical challenges of adolescent diabetes management and require targeted policy and clinical interventions.

The stigma and misconceptions about diabetes described by participants highlight the need for community education initiatives that address cultural beliefs and promote understanding of diabetes as a manageable medical condition rather than a personal failing or contagious disease.

### 3.3. Clinical and Policy Implications

Based on these findings, several important implications emerge:

1. Culturally Responsive Care Models: healthcare providers need training in culturally sensitive diabetes education that acknowledges Palestinian cultural practices around food, religious observances, and family dynamics.
2. Family-Centered Interventions: given the central role of family support, diabetes education, and management programs should actively involve family members and provide them with appropriate resources and training.
3. Community Education Programs: public awareness campaigns are needed to combat stigma and misconceptions about diabetes within Palestinian communities.
4. Peer Support Initiatives: the expressed desire for peer connections suggests that structured peer support programs could significantly benefit Palestinian youth with diabetes.
5. Healthcare System Strengthening: policy efforts must address systemic barriers, including medication supply chains, specialist training, and healthcare accessibility across the West Bank.
6. Spiritual Care Integration: healthcare providers should acknowledge and incorporate patients' spiritual and religious resources into diabetes care planning.

### 3.4. Study Strengths and Limitations

This study provides the first in-depth qualitative exploration of diabetes management experiences among Palestinian youth, offering important insights for healthcare providers and policymakers. The use of content analysis allowed for systematic identification of key themes while maintaining the richness of participants' experiences.

However, several limitations must be acknowledged. The findings, derived from the specific context of the West Bank, may not be directly transferable to Palestinian youth in other regions or to other populations. The sample size, while appropriate for qualitative research, limits generalizability. Additionally, the cross-sectional nature of the study provides a snapshot of experiences at one point in time rather than tracking how coping strategies develop over time.

The focus on the West Bank means these findings may not fully represent experiences of Palestinian youth living in Gaza, Jerusalem, or diaspora communities, where different political, economic, and healthcare factors might substantially influence diabetes management experiences.

### 3.5. Future Research Directions

Future research should explore the effectiveness of culturally tailored interventions based on these findings. Longitudinal studies tracking the development of coping strategies over time would provide valuable insights into how adaptation occurs. Comparative studies with Palestinian youth in different contexts (Gaza, diaspora communities) could illuminate the role of environmental factors in diabetes management experiences.

Additionally, research focusing on family members' experiences and the development of family-centered intervention models would complement these findings and support comprehensive care approaches.

## 4. Conclusion

This study illuminates the complex reality of living with type 1 diabetes as a Palestinian youth in the West Bank. The findings demonstrate that effective diabetes management in this population requires understanding and addressing not only medical needs but also cultural, social, and systemic challenges that shape daily life.

The remarkable resilience demonstrated by these young people, their reliance on family support systems, and their integration of spiritual resources into coping strategies provide important insights for healthcare providers and policymakers. Their experiences underscore the critical need for healthcare approaches that are culturally responsive, family-centered, and sensitive to the broader context of life in Palestine.

These findings contribute to the growing body of literature on chronic illness management in conflict-affected and resource-limited settings while amplifying the voices of Palestinian youth themselves. Their stories of adaptation, resilience, and hope, despite significant challenges, offer valuable lessons for supporting young people with diabetes in similar contexts worldwide.

Most importantly, this research provides evidence to support the development of more responsive and effective healthcare approaches that honor both the medical needs and cultural values of Palestinian youth with diabetes, ultimately improving their health outcomes and quality of life.

## Figures and Tables

**Table 1:** Participant demographic characteristics (*N* = 18).

Characteristic	*n* (%) or Mean ± SD
Age (years)	15.2 ± 1.8
12–14 years	6 (33.3)
15–16 years	7 (38.9)
17–18 years	5 (27.8)
Gender	
Female	9 (50.0)
Male	9 (50.0)
Duration of T1DM (years)	4.3 ± 2.1
6 Months–2 years	4 (22.2)
2–5 Years	8 (44.4)
>5 Years	6 (33.3)
Location	
Nablus	8 (44.4)
Jenin	5 (27.8)
Ramallah	3 (16.7)
Hebron	2 (11.1)
Family income	
<2000 NIS/month	12 (66.7)
2000–4000 NIS/month	4 (22.2)
>4000 NIS/month	2 (11.1)

## Data Availability

The data that support the findings of this study are available on request from the corresponding author. The data are not publicly available due to privacy or ethical restrictions.

## Supporting Information

Supporting Information 1 The following supporting information is available for this study: Supporting Information S1. Completed COREQ (Consolidated Criteria for Reporting Qualitative Research) checklist.

Supporting Information 2 Supporting Information S2. Expanded participant demographics, thematic overview with key quotes, coping strategies, barriers, and methodological details.
