# Supplementary material for: Lived Experiences of Coping With Type 1 Diabetes Among Palestinian Youth: A Qualitative Study
Source: Pediatr Diabetes. 2025 Nov 2;2025:9014326. doi: 10.1155/pedi/9014326 (PMC12597229; doi:10.1155/pedi/9014326)
Supplement: Supporting Information 2 — Supporting Information S2. Expanded participant demographics, thematic overview with key quotes, coping strategies, barriers, and methodological details. [file 9014326.f2.docx]

**Participant Demographics**
Demographic profile of the 18 participants:

- **Age (Mean):** 15 years
- **Gender Distribution:**
  - Male: 50% (n=9)
  - Female: 50% (n=9)
- **Inclusion Criteria:**
  - Age: 12–18 years
  - Duration of T1DM diagnosis: >6 months
- **Location:** West Bank, Palestine
- **Recruitment Sites:** Diabetes clinics and youth support networks
- **Study Period:** 11 May 2025 to 10 June 2025.

**Thematic Overview of Lived Experiences**
Summary of major themes and subthemes identified:

- **Theme 1: Social and Cultural Pressures**
  - Subthemes: Stigma, dietary expectations, social isolation
  - Key Quotes:
    • “They think diabetes is contagious.”
    • “Celebrations have a lot of sugary foods—it’s hard to say no.”
- **Theme 2: Support Systems**
  - Subthemes: Family involvement, healthcare access, peer support
  - Key Quotes:
    • “My family provides emotional support.”
    • “It’s difficult to access insulin due to shortages.”
- **Theme 3: Emotional Coping**
  - Subthemes: Resilience, anxiety management, faith-based strategies
  - Key Quotes:
    • “I’ve learned to accept that diabetes is part of my life.”
    • “I use prayer to find peace.”
- **Theme 4: Daily Self-Management**
  - Subthemes: Insulin adherence, dietary planning
  - Key Quotes:
    • “I carry my insulin everywhere.”
    • “I’ve learned to read food labels.”

**Coping Strategies Identified**
Strategies frequently reported by participants (n=18):

| **Coping Strategy** | **% of Participants Reporting** |
| --- | --- |
| Parental/family support | 100% |
| Religious or spiritual coping | 72% |
| Peer-based support | 61% |
| Structured insulin and meal planning | 89% |
| Avoidance/distraction (e.g., hobbies) | 67% |

**Barriers to Effective Diabetes Management**
Reported challenges affecting disease management:

- **Healthcare Access:**
  • Shortages of insulin and supplies (reported by 8/18)
  • Limited diabetes education at clinics
- **Social Factors:**
  • Pressure to consume traditional foods
  • Stigma and misunderstanding by peers and elders
- **Psychological Stressors:**
  • Anxiety about complications
  • Fear of hypoglycemic episodes in public

**Ethical and Methodological Information**

- **Ethics Approval:** Arab American University IRB (Code: R-2025/A/29/N)
- **Informed Consent:** Obtained from all participants and guardians (for minors)
- **Interview Duration:** 45–60 minutes per session
- **Data Collection Period:** Not explicitly stated (assumed recently, based on IRB date)
- **Data Handling:** Audio-recorded, anonymized, stored securely
- **Follow-up Interviews:** Conducted with 5 participants for saturation and member checking
- **Analysis Method:** Thematic analysis using phenomenological approach
- **Data Saturation Achieved:** After the 15th interview (total interviews = 18)
- **Data collection:** 11 May–10 June 2025.

**Emotional Well-being Insights**

- **Common Emotional Responses:**
  • Anxiety (83%)
  • Resilience (78%)
  • Religious acceptance (72%)
  • Frustration or burnout (61%)
- **Mental Health Support:**
  • On-call counselor during interviews
  • Participants emphasized need for more psychological support in care settings

**Practical Implications Suggested by Participants**

- Youth-centered educational initiatives
- Peer support groups in clinics
- More access to diabetes specialists and supplies
- Cultural sensitivity in dietary guidance
- Greater public awareness campaigns to reduce stigma
